# Empowering people affected by penile cancer: towards a model for supportive self-management

**DOI:** 10.1038/s41443-025-01042-5

**Published:** 2025-03-19

**Authors:** Catherine Paterson, Wayne Earle, David Homewood, Justin Chee, Henry Yao, Shomik Sengupta, Oluwaseyifunmi Andi Agbejule, Reegan Knowles, Carolyn Ee, Theo Niyonsenga, Ian D. Davis

**Affiliations:** 1 Flinders University, Caring Futures Institute, Adelaide, SA, Australia; 2 Central Adelaide Local Health Network, Adelaide, SA, Australia; 3 Check Your Tackle, Consumer Not-for-Profit Organisation, Melbourne, VIC, Australia; 4 Department of Urology, Western Health, Melbourne, VIC, Australia; 5 Department of Surgery, Western Precinct, University of Melbourne, Parkville, VIC, Australia; 6 International Medical Robotics Academy, Melbourne Australia, Parkville, VIC, Australia; 7 Eastern Health Clinical School, Monash University Faculty of Medicine, Nursing and Health Sciences, Parkville, VIC, Australia; 8 Eastern Health, Parkville, VIC, Australia; 9 Faculty of Health, University of Canberra, Canberra, ACT, Australia

**Keywords:** Quality of life, Therapeutics

## Abstract

Improvements in the quality of penile cancer management are difficult due to the rarity of the condition and a limited evidence base for treatment decisions. Penile cancer and some of its highly morbid treatments can cause profound psychosexual and physical effects that negatively impact quality of life. Multidisciplinary interventions are required to equip patients with the support necessary to manage their emotional, physical, work, and lifestyle challenges to optimize health, well-being, and recovery. This paper outlines a model of supported self-management, which is a novel model of care for people with penile cancer to mitigate disease and treatment morbidity.

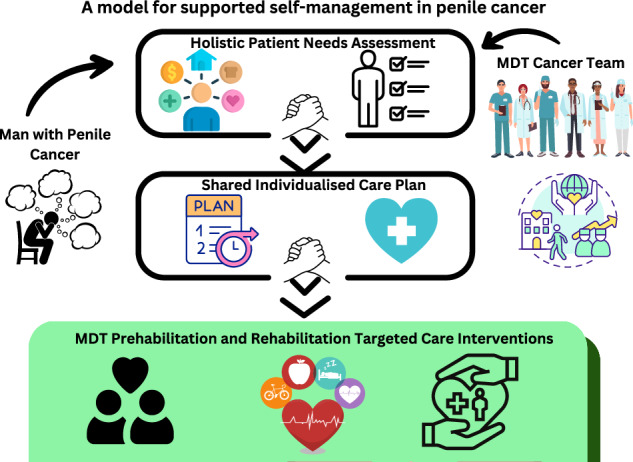

## Introduction

Penile cancer is a rare malignancy that can have an aggressive natural history, and often requires highly morbid surgical interventions [1]. Penile cancer has varying incidence rates globally. In high-income countries, the standardised incidence has been reported at 0.94/100,000 in Europe and 0.5/100,000 in the USA [2]. In contrast, incidence rates in low- to middle-income countries have been reported as high as 0.7–3.0/100,000 in India, 8.3/100,000 in Brazil, and even higher in other parts such as Africa [3, 4]. Due to the rarity of the disease and the variability of global distribution, the options for clinical management and access to supportive cancer care are inequitable and fragmented at the individual (each patient), meso (health system), and macro (policy-making) level [5–7]. Moreover, recent evidence demonstrates guideline knowledge is suboptimal with inconsistent clinician adherence [8, 9] leading to unwanted variations in patient outcomes and subsequent requirements for supportive care.

Supportive care is a person-centred approach to the delivery of cancer services for those affected by cancer to meet their emotional, physical, social, spiritual, and informational needs during diagnosis, treatment, or follow-up phases of care. These needs include issues of health promotion, preventive care, survivorship, palliation, and bereavement care [5, 10]. A diagnosis of penile cancer and its associated highly morbid treatments presents numerous challenges that have been largely unmet in routine service delivery as evidenced by a recent systematic review [5]. Penile cancer causes significant distress due to physical issues (fatigue, genital pain, urinary and sexual dysfunction, scars, stenosis and bleeding, lymphoedema, sleep problems, mobility limitations, reduced strength, frequent infections, and impaired wound healing) [11–15], and psychosocial morbidity (anxiety, depression, suicidal ideation, sadness, lowered self-esteem, post-traumatic stress disorder, altered body image and masculine identity, isolation, fear of death and dying) [13, 16, 17]. Men living with penile cancer receive very limited, if any, supported self-management interventions or pre-and/or rehabilitative models of care [5, 18, 19]. This discussion article aims to provide a new direction and model for delivering person-centred care to optimize supported self-management for men affected by penile cancer [5, 6].

## Supported self-management

It is necessary to distinguish the concepts of self-care, self-management, and supported self-management in order to facilitate the further development of coherent and consistent research, policy, and practice recommendations and address existing care shortcomings among men with penile cancer [5, 6]. The World Health Organization defines self-care as “the activities individuals, families, and communities undertake with the intention of enhancing health, preventing disease, limiting illness, and restoring health” [20]. This description reflects that self-care skills and knowledge stem from lay experience and suggests that self-care is a part of daily living for all. Whereas self-management refers to an individual’s ability to manage the physical, psychological, and social consequences, symptoms, treatment regimes, and lifestyle changes inherent in living with a long-term condition, such as cancer [21, 22]. The concept of self-management is associated with the management of long-term conditions by patients at home unsupervised by healthcare professionals [23]. On the other hand, *supported* self-management is a patient-centered collaborative approach to empowerment, patient activation, and education that extends the role of healthcare professionals beyond the delivery of information and traditional patient education [22, 24, 25].

In *supported* self-management, healthcare professionals actively collaborate with patients to facilitate increased levels of confidence and self-efficacy and engage in shared decision-making to deliver improved self-management and better quality-of-life outcomes. Supported self-management provides individualised patient support to enhance self-management behaviours, improve problem-solving capabilities, while promoting an active collaboration with the cancer healthcare team [26]. The supported self-management model of care has been highlighted as a critical component to ensure patient well-being, particularly in long-term conditions, like cancer, and the increased use of non-pharmacological approaches to symptom management [27]. See Table 1 for characteristics of self-care, self-management, and supported self-management in cancer.

**Table 1:** Characteristics of self-care, self-management, and supported self-management in cancer.

Concept	Who is involved	Goals or targets	What is involved
Self-care	• Universal for all • Ranges from the individual person or patient to families inclusive of communities in collaboration with healthcare professionals and healthcare systems • However, healthcare professionals need not be involved	• Optional to have goals and targets • Examples: Prevention of disease and accidents, reduce illness and restoration of health • Improvement in the existing state of health, which may or may not be associated with a long-term condition • Changes in lifestyle, maintenance of optimal levels of health • Recovery from minor ailments and following discharge from hospital	• May include doing nothing • Taking responsibility for health for self, children, family and helping others • Asserting control • Managing emotions • Goal attainment and behavioural change
Self-management	• More focused on cancer networks in health • Patients, peers, healthcare practitioners and support networks • Includes healthcare professional as collaborator with the person with the cancer	• Desirable to have goals and targets • Minimization of the impact of cancer on physical health status and functioning; coping with the psychological effect of cancer and treatments • Minimization of symptom frequency, bother, burden, and distress • Patients participate in decision-making about treatment, gaining a sense of control over their lives • Initiation or maintenance of access to health services and practitioners • Targeting change in behaviour, existing and new behaviours	• Active participation by a person with cancer • Symptom management • Behavioural tasks • Individual or group peer tasks • Medical management • Self-regulation/self-monitoring of condition • Lifestyle change and education
Supported self-management	• Highly focused and complex networks • Patients, practitioners and the healthcare system • May require a refocus of health practitioner activity	• Essential to have goals and targets • Service development and cancer disease management improvement, including the provision of supported self-management, shared decision support, delivery system redesign, and clinical information systems (including capture patient-reported outcome measures) • Development of new skills in practitioners (e.g. problem-solving and goal-setting), with the patient as a key resource • Patient empowerment, activation and education • Increasing self-management skills • Cognitive symptom management, positive behaviour changes	• Emphasizes distinct collaboration between patient, healthcare practitioner and healthcare system, in standardized, programmatic interventions to improve self-management behaviour

## Supported self-management in penile cancer

The multifaceted symptoms and effects associated with penile cancer present significant management challenges to the patient and healthcare professional, across the entire cancer care continuum, from diagnosis, to survivorship, and palliative and end-of-life care [5, 17]. Urinary and sexual dysfunction, along with other psychosocial and physical issues, can significantly impact the quality of life for people affected by penile cancer. Clinical management approaches, such as organ-preserving surgery where feasible [28, 29], may help improve cosmetic outcomes and sexual function. Long-term management of the psychosocial and physical symptoms associated with penile cancer often involves addressing behavioral risk factors (e.g., depression, anxiety, body image concerns, urinary dysfunction, fatigue) through undertaking self-management behaviours (e.g., undertaking specific exercises, engaging in cognitive therapy techniques). Examples of self-management strategies for penile cancer-related symptoms are presented in Table 2.

**Table 2:** Examples of supported self-management in penile cancer.

Penile Cancer-related Symptom	Evidence-based self-management strategies	Supported self-management
**Depression/Anxiety**	Cognitive Behavioural Therapy (CBT) techniques, such as thought journaling and relaxation exercises Mindfulness-based interventions Relaxation therapies [43]	Provide access to mental health resources, such as CBT apps or support groups. - Regular follow-ups to assess mental health status.
**Urinary Dysfunction**	Shortened penis (in high BMI patients) or loss of navicular fossa, or perineal urethrostomy requires urination modification (sitting down or using funnels) Intermittent urethral dilatation for the prevention of strictures post-radiotherapy [44] Pelvic floor muscle exercises to improve stress incontinence [45] Urge incontinence requiring bladder retraining or Transcutaneous tibial nerve stimulation (TTNS) may be used as some men (especially older ones) for concurrent overactive bladder [46]	Guidance from a physical therapist specializing in pelvic health. - Use of reminder tools (apps, charts, aids) to promote regular exercise practice.
**Pain**	Mindfulness-based stress reduction (MBSR) and pain management techniques, such as guided imagery and progressive muscle relaxation [47]	Offer training sessions or workshops on MBSR techniques. - Provide access to pain management specialists or support groups.
**Body Image Issues**	CBT Participation in body-positive support groups and self-compassion exercises Couples-based interventions [48]	Provide access to counseling services that focus on body image. - Encourage involvement in social support networks or peer-led groups.
**Sexual Dysfunction**	Psychosocial counselling Use of pro-erectile agents and devices Sexual rehabilitation exercises and open communication with partners about needs and concerns [49]	Facilitate access to sexual health specialists and counseling. - Provide educational materials and workshops for patients and their partners.
**Social Isolation**	Staying connected Maintaining a routine Getting outside Reconnecting with interests and hobbies Acknowledging feelings [50, 51]	Target opportunities for community-based support, signpost to psychosocial groups/rehab; friendship enrichment clubs; Experimental study on social isolation and interventions; One-on-one interventions; Volunteering.
**Lymphedema**	Early use of fitted compression devices, and a pre-emptive referral to a lymphedema specialist [52, 53]	Offer referral to lymphedema specialist for treatment

Patient engagement in self-management behaviours and strategies can be complex, as it can require individuals to recognize, track, self-monitor, self-report, and apply problem-solving skills for their penile cancer-related symptoms alongside any other comorbid conditions; tasks with which most patients are not familiar. For example, patients undertaking intermittent self-dilation for urethral stricture disease following radiotherapy or performing self-examination of penis and inguinal lymph nodes to look for recurrence of cancer in the setting of penile sparing therapy. Furthermore, factors such as the severity of symptoms, socioeconomic status, mental health, cognitive ability, age, physical performance, and other situational influences can complicate the capabilities of patients to self-manage. The severity of treatment options and associated consequences of urinary and sexual dysfunction, coupled with psychological effects and altered perceptions of masculinity, underscores the need for anticipatory and timely supported self-management models of care delivery to help patients navigate the complexities associated with the self-management of penile cancer side effects [6, 13, 17]. To the best of our knowledge, there have been no reported studies [5, 6, 19] in supported self-management interventions in penile cancer. However, in other genitourinary cancer patient groups who experience issues such as urinary and sexual dysfunction (which are common to penile cancer), a nurse-led model of supported self-management, safely embedded in the Multidisciplinary Team (MDT), was found to improve supportive care and patients’ ability to self-manage [30, 31].

**Fig. 1:**
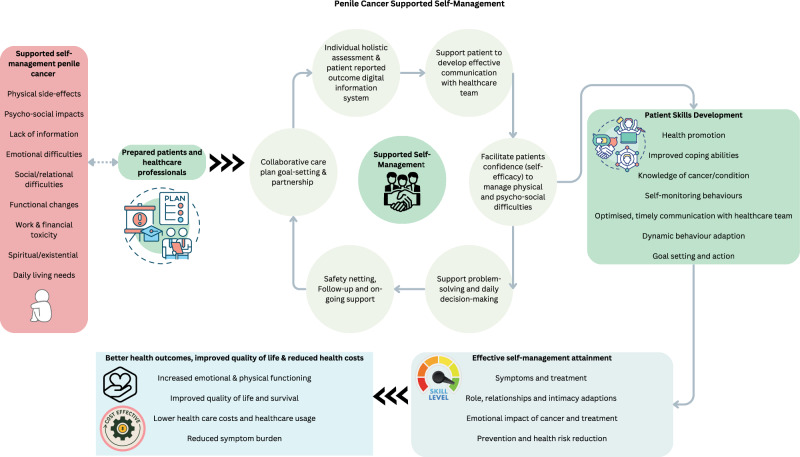
Penile Cancer Supported Self-Management.

Effective supported self-management requires a multidisciplinary approach tailored to individual needs (Fig. 1). In fact, penile cancer provides an exceptionally compelling case for supported self-management programs not just for centralised referral cancer centres [32–34] but equity in quality care for all individuals. The multidisciplinary team’s involvement should be tailored to the individual across the cancer care continuum [35] which includes surgeons, oncologists, radiation oncologists, pathologists, radiologists, specialist nurses, physiotherapists, exercise physiologists, health psychologists, social workers, radiation therapists, dieticians, lymphedema practitioners, palliative care practitioners, general practitioners, and relationship therapists who are actively engaged with the model of supported self-management. Supported self-management in other cancer types has been shown to increase emotional and physical functioning, improve quality of life and survival, reduce healthcare costs and usage, and reduce symptom burden [36–38]. People with penile cancer are particularly vulnerable and as healthcare professionals aspire to greater personalized care and treatment it is essential to enable those affected by penile cancer to be active contributors to the self-management of their disease and health [1].

## Outlook and future

First, there is a need to explore and clearly define the self-management behaviors and factors associated with improved symptom management for patients with penile cancer. Additionally, definition of symptom clusters—groups of related symptoms that occur together—can provide insights into how self-management strategies might address multiple symptoms simultaneously. This knowledge in turn may allow efficiencies in the design of interventional strategies and help tailor self-management interventions to enhance overall symptom control and quality of life for men with penile cancer. While the delivery of supported self-management is highly attainable in centralised penile cancer services, for example, in North America, Europe and Australia, it may not be possible in all parts of the world particularly in low- and middle-income countries where the development of cancer nursing and cancer services requires significant support from the international cancer partners, as evidenced by recent research [39]. Creating meaningful change in penile cancer management in such countries will require policymakers, government officials, and international cancer organizations to continue to work together to support cancer control in light of the current and projected limited resources and barriers in cancer diagnosis and management [40, 41].

## Conclusion

Current knowledge is limited regarding the implementation, effectiveness, and impact on patient outcomes of supported self-management programs in penile cancer. Supported self-management has been shown in other settings to be a powerful lever to achieving personalized high-quality care by facilitating patients’ skills development to attain effective self-management capabilities [42]. This approach requires a refocus and change of healthcare professional activity to create a distinct collaborative partnership with patients to enable patient empowerment, activation, and education in self-management. Healthcare professionals and healthcare systems must adapt to better meet the needs of people with this rare cancer. Champions in organisations across the globe, particularly in centralised referral cancer centres are needed to lead the way in high-quality and consistent self-management support to improve the lives of men with penile cancer.
